# Overexpression of *luxS* Promotes Stress Resistance and Biofilm Formation of *Lactobacillus paraplantarum* L-ZS9 by Regulating the Expression of Multiple Genes

**DOI:** 10.3389/fmicb.2018.02628

**Published:** 2018-11-12

**Authors:** Lei Liu, Ruiyun Wu, Jinlan Zhang, Pinglan Li

**Affiliations:** ^1^Beijing Advanced Innovation Center for Food Nutrition and Human Health, College of Food Science and Nutritional Engineering, China Agricultural University, Beijing, China; ^2^Key Laboratory of Functional Dairy, China Agricultural University, Beijing, China

**Keywords:** *Lactobacillus paraplantarum* L-ZS9, *luxS*, stress resistance, biofilm, RNAseq

## Abstract

Probiotics have evoked great interest in the past years for their beneficial effects. The aim of this study was to investigate whether *luxS* overexpression promotes the stress resistance of *Lactobacillus paraplantarum* L-ZS9. Here we show that overexpression of *luxS* gene increased the production of autoinducer-2 (AI-2, quorum sensing signal molecule) by *L. paraplantarum* L-ZS9. At the same time, overexpression of *luxS* promoted heat-, bile salt-resistance and biofilm formation of the strain. RNAseq results indicated that multiple genes encoding transporters, membrane proteins, and transcriptional regulator were regulated by *luxS*. These results reveal a new role for LuxS in promoting stress resistance and biofilm formation of probiotic starter.

## Introduction

Over the past three decades, probiotic potential of some bacteria has been well-recognized. The beneficial effects of probiotic bacteria are mainly associated with the maintenance of a healthy gut microbiota, an improvement of gut resilience, the modulation of lactose intolerance, bowel function, and gastrointestinal (GI) comfort, and diarrhea prevention and symptom alleviation (Licciardi et al., 2010; Waitzberg et al., 2015; Wright et al., 2015; Kich et al., 2016; Mokoena et al., 2016). Probiotic food products, including probiotic powders and probiotic fermented food, account for a significant part of the functional foods in market (60–70% of the total functional food in market; García-Ruiz et al., 2014). Notably, to exert positive health effects, they have to colonize in the certain region of gastrointestinal tract and maintain themselves in certain numbers. The microorganism should survive adverse conditions during processing, storage, and the gastrointestinal transit. Therefore, it is important to enhance the stress resistance of probiotic bacteria. Although traditional researches focus on the development of protection materials and high density fermentation (Mamvura et al., 2011; Gbassi and Vandamme, 2012; Ortakci et al., 2012; D'Orazio et al., 2015; Ilango et al., 2016; Champagne et al., 2017), there is a great prospect to promote the stress resistance of probiotic bacteria by genetic engineering based on their molecular physiology.

Various bacteria use quorum sensing (QS) to communicate with each other. Autoinducer-2 (AI-2) is the universal language for intra-species and inter-species communication. Biosynthesis of AI-2 involves a three-step reaction, which is part of methionine catabolism cycle. Homolog of *luxS* which encodes the protein responsible for AI-2 production has been found in a wide range of both Gram-positive and Gram-negative bacteria, with 17% of the phylum Bacteroidete and 83% of the Firmicutes predicted to have the homolog. This implies that some of the non-pathogenic or beneficial bacteria also use this “universal” and “common” signaling system to regulate their own behavior, including stress resistance and biofilm formation. However, the universal *luxS*-mediated quorum sensing using the autoinducer-2 (AI-2) signal is present in a wide variety of bacteria with only sparse information on probiotic lactobacilli (Yeo et al., 2015). It has been reported that AI-2-dependent autoaggregation enhances bacterial stress resistance and promotes biofilm formation of *Escherichia coli* (Laganenka et al., 2016). AI-2 signaling is also involved in the regulation of stress-related genes of *Deinococcus radiodurans* and triggers the oxidative stress response in *Mycobacterium avium* (Geier et al., 2008; Lin et al., 2016). As for lactic acid bacteria (LAB), it has been reported that *Lactobacillus* spp. use AI-2 signaling to respond to environmental stress and to regulate growth and metabolism (Park et al., 2016).

*Lactobacillus paraplantarum* L-ZS9 is a strain originally isolated from fermented sausage and proved to produce class II bacteriocins to inhibit the growth of pathogenic bacteria (Liu and Li, 2016; Zhang et al., 2016). It has a potential to be used as a probiotic and fermentation starter. In this study, we examined whether *luxS* overexpression enhances stress resistance of *L. paraplantarum* L-ZS9 and which genes are regulated by *luxS* overespression. We show that the effect of *luxS* on stress resistance including heat-, acid-, and bile-tolerance and biofilm formation of this strain. Furthermore, we identified multiple genes encoding transporters, membrane proteins, and transcriptional regulator regulated by *LuxS*. These results provide new insights into the regulation of stress resistance of probiotic starter and the molecular mechanisms.

## Materials and methods

### Bacterial strains and growth conditions

*L. paraplantarum* L-ZS9 originally isolated from fermented sausage was grown at 37°C in de Man-Rogosa-Sharpe (MRS) broth (Bridge, Beijing) or MRS-Agar aerobically. *Escherichia coli* DH5α (Takara, Dalian) was grown in Lennox broth (LB) or LB agar at 37°C. *Escherichia coli* DH5α containing pMG76e or *luxS*-pMG76e plasmid was cultured in LB medium containing 200 μg/mL erythromycin. *L. paraplantarum* L-ZS9 containing pMG76e or *luxS*-pMG76e plasmid was cultured in MRS broth or MRS-Agar containing 3 μg/mL erythromycin. Vibrio harveyi BB170 (sensor1- sensor2+) and BB152, kindly provided by Professor Xiangan Han (Shanghai Veterinary Research Institute, CAAS, Shanghai, China), were grown in Marine Broth 2216 (Difco Co., USA) or Autoinducer Bioassay (AB) medium. BB170 is an AI-2 biosensor strain. BB152 was used as a positive control for AI-2 production.

### Construction of plasmids and bacterial strains

Chromosomal DNA of *L. paraplantarum* L-ZS9 was isolated using TIANamp Bacteria DNA Kit (Tiangen Biotech, Beijing). Gene *luxS* was amplified by PCR using primers 5′-TGCTCTAGAATGGCTAAAGTAGAAAGTTT-3′ (forward) and 5′-CCGCTCGAGCTATTCAACGACTTTGCGAA-3′ (reverse) containing restriction sites XbaI and XhoI. The PCR product was cloned into pMD18T vector (Takara, Dalian) to construct vector *luxS*-pMD18T. Subsequently, *luxS*-pMD18T vector (P_lac_ as the promoter) and pMG76e vector (P_32_ as the promoter) (kindly provided by Professor Shangwu Chen, China Agricultural University) were digested with fastDigest enzymes XbaI and XhoI (Thermo Scientific) at 37°C for 5 min to obtain *luxS* gene fragment and dual-enzyme digested linearized pMG76e plasmid. The *luxS* gene fragment was inserted into the linearized pMG76e plasmid using Rapid DNA Ligation Kit (Thermo Scientific). The ligation product was transformed into *E. coli* DH5α and verified by PCR using primers 5′-TTCGGTCCTCGGGATATG-3′ (forward) and 5′-CTGTCTTGGCCGCTTCAA-3′ (reverse). *luxS*-pMG76e and empty pMG76e plasmids were electro-transformed into *L. paraplantarum* L-ZS9 competence cells. Recombinant strains were selected with erythromycin (3 μg/mL) and verified by PCR.

### Real-time quantitative PCR (qRT-PCR)

Recombinant strains *luxS*-pMG76e-L-ZS9 and pMG76e-L-ZS9 and the wild-type strain L-ZS9 were cultured for 8 h achieve logarithmic phase. Total RNA was extracted from these cells using TRIzol reagent (Invitrogen) according to the manufacture's protocol. RNA quality was determined by A_260_/A_280_, A_260_/A_230_, and electrophoresis. Isolated RNA was transcribed into single-stranded cDNA using TUREscript 1st Strand cDNA Synthesis Kit (Aidlab Biotechnologies Co., Ltd). qRT-PCR was performed using a SYBR Green assay kit (Tiangen) and the 7500 Fast Real-Time PCR system (Applied Biosystems). Primers were designed using Primer 3 Input. 16S rRNA was used as an internal reference. The relative expression of *luxS* was calculated by using the 2^−ΔΔ*CT*^ method according to Livak and Schmittgen (2001).

### AI-2 detection

Recombinant strains *luxS*-pMG76e-L-ZS9 and pMG76e-L-ZS9 and the wild-type strain L-ZS9 were cultured in 12% (w/v) skim milk medium without erythromycin. At 2, 4, 6, 8, 10, 12, 14, 16, 18, 20, 22, and 24 h, cells were centrifuged at 12,000 g at 4°C for 10 min. The cell-free culture fluid (CF) was obtained by filtering the supernatant through a 0.22-μm filter (Millipore, Bedford, MA, USA) and adjusting the pH to 7.0. The reporter strain *V. harveyi* BB170 was diluted 1:5,000 in AB medium and the CF sample was added to the diluted BB170 culture at 1:10 (v/v). The mixture was incubated at 28°C for 5 h. One hundred microliters of aliquots were added to white, flat-bottomed, 96 well plates (Thermo Labsystems, Franklin, MA, USA) to detect AI-2 activity. The CF from BB152 and DH5α were used as the positive and negative control, respectively. Luminescence values were measured with a Tecan GENios Plus microplate reader in luminescence mode (Tecan Austria GmbH, Grodig, Austria).

### Growth curves

Recombinant strains *luxS*-pMG76e-L-ZS9 and pMG76e-L-ZS9 and the wild-type strain L-ZS9 were cultured in MRS without erythromycin. At 3, 6, 9, 12, 15, 18, 21, 24, 27, 30, 33, and 36 h, the cell densities were determined by measuring OD_600_ using a UV-1800 Spectrophotometer. Measurements were carried out in triplicate.

### Biofilm formation

Crystal violet (CV) staining was used to quantify biofilm formation by recombinant strains *luxS*-pMG76e-L-ZS9 and pMG76e-L-ZS9 and the wild-type strain L-ZS9. Briefly, overnight cultures were diluted to an optical density of 0.1 at 600 nm. Two hundred microliters of diluted culture were transferred to a 96-well plate (Corning, NY, USA). After incubation at 37°C for 36 h, the wells were washed gently three times with phosphate-buffered saline (PBS), stained with 0.1% CV for 30 min at room temperature, rinsed with distilled water and air-dried. One hundred microliters of 95% ethanol was added to each well to dissolve the CV. The optical density at 595 nm was determined using a Synergy 2 microplate reader (Biotek, Winooski, VT, USA).

### Stress resistance assay

Recombinant strains *luxS*-pMG76e-L-ZS9 and pMG76e-L-ZS9 and the wild-type strain L-ZS9 were cultured in MRS without erythromycin for 8 h to logarithmic phase. Cells were centrifuged at 5,000 g at 4°C for 5 min and washed with PBS for three times. Cells were treated at 80°C for 3 min. Survived cells were determined by flat colony counting method to assess the heat resistance. To compare acid- and bile- resistance, cells were centrifuged at 5,000 g for 5 min and then resuspended in PBS (pH 2.8) or PBS containing bile salt (0.2%, w/v). After 30 min, the survived cells were determined by flat colony counting method. Cell heat-, acid-, and bile- resistance was assessed by cell survival rates. The survival rates were calculated as follows: survival cell number/initial cell number × 100%. The initial cell number was 1 × 10^9^.

### Total RNA extraction and sequencing

Recombinant strains *luxS*-pMG76e-L-ZS9 and pMG76e-L-ZS9 were cultured in MRS with erythromycin for 8 h to logarithmic phase. Total RNA (3 independent extractions for each strain) was isolated using the TRIzol reagent (Ambion) according to the manufacturer's instructions and treated with DNase I to remove DNA. The MICROBExpress Kit (Ambion) was used to deplete rRNA from total RNA. Libraries were constructed using NEBNext1Ultra™ Directional RNA Library Prep Kit (Illumina). One hundred bp single end sequence reads were generated using the Illumina HiSeq 4000 platform at the Vienna Biocenter Campus Science Support Facility. Sequencing adapter removal was performed with cutadapt. Mapping of the samples against the *L. paraplantarum* L-ZS9 reference genome (NCBI accession number CP013130) was performed with Segemehl with default parameters. Reads mapped to be rRNA or tRNA were discarded from all data and ignored for all follow up analysis. The mapped sequencing data were prepared for visualization using the ViennaNGS tool box and visualized within the UCSC Genome Browser. Differential gene expression analysis was performed with DESeq (version 1). All genes with a fold change >2.0 and a multiple testing adjusted *p*-value below 0.05 were considered to be significantly modulated. All RNA-seqs were performed triple biological replicates per sample.

### Statistical analysis

The data were analyzed by one-way analysis of variance (ANOVA) using Graphpad prism 5.0. Data are presented as means ± SEM. *P* < 0.05 were considered significant.

## Results

### Overexpression of *luxS* gene promotes AI-2 production by *L. paraplantarum* L-ZS9

**Figure 1:**
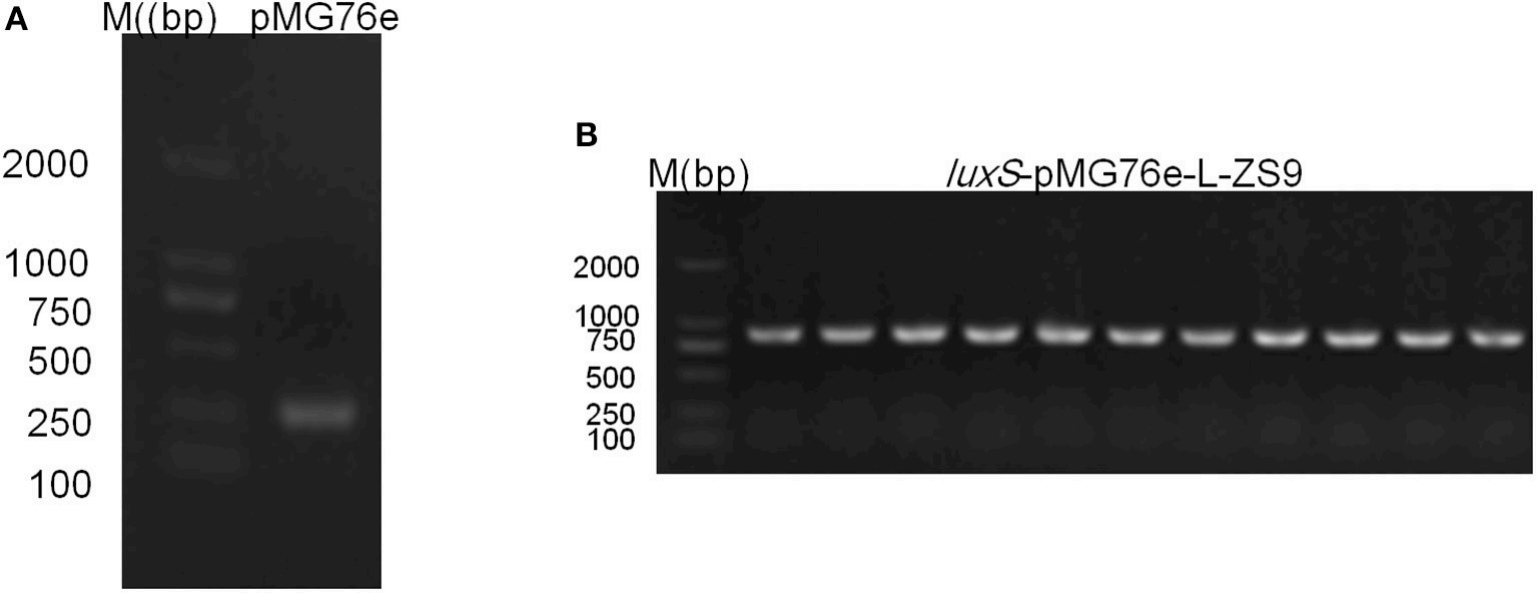
Agrose gel electrophoresis of PCR validation result of pMG76e-L-ZS9 **(A)** and *luxS*-pMG76e-L-ZS9 **(B)** strains using primers 76e-F/R.

**Figure 2:**
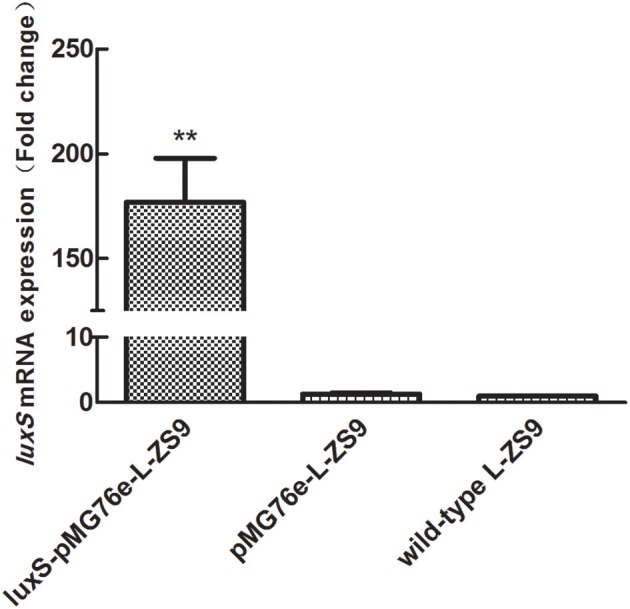
*luxS* mRNA expressions of pMG76e-L-ZS9, *luxS*-pMG76e-L-ZS9 and wild-type strains. Data are presented as mean ± SEM. *n* ≥ 3. **p < 0.01 compared with the negative control.

Recombinant strains *luxS*-pMG76e-L-ZS9 and pMG76e-L-ZS9 were constructed from parent strain *L. paraplantarum* L-ZS9 and identified by PCR (Figure 1). In order to validate the overexpression of *luxS* gene, qRT-PCR was carried out to analyze the relative mRNA level of *luxS* in *luxS*-pMG76e-L-ZS9, pMG76e-L-ZS9, and wild-type L-ZS9 cells. As shown in Figure 2, the *luxS* mRNA expression in *luxS*-pMG76e-L-ZS9 cells were up-regulated about 177-folds compared to that in L-ZS9 cells, but *luxS* transcription in pMG76e-L-ZS9 cells was comparable to wild type cells.

**Figure 3:**
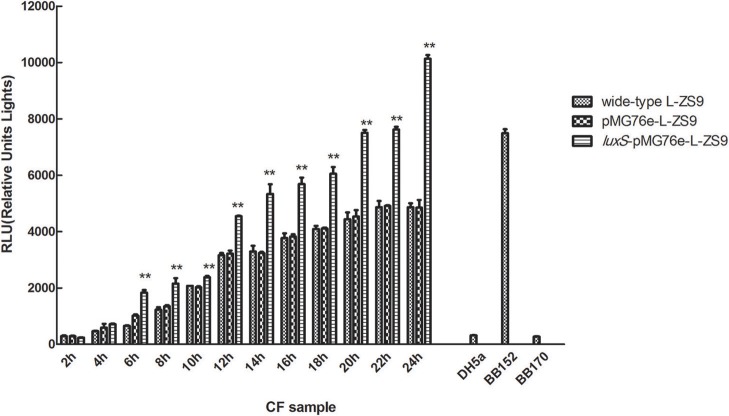
AI-2 activity in cell-free culture fluids (CF) of pMG76e-L-ZS9, *luxS*-pMG76e-L-ZS9 and wild-type strains. *V. harveyi* BB152 served as a positive control and *E. coli* DH5α as a negative control. Data are presented as mean ± SEM. *n* ≥ 3. **p < 0.01 compared with the negative control.

*Luxs* gene encodes an enzyme involved in the metabolism of S-ribosylhomocysteine, finally leading to the production of AI-2. AI-2 activities were measured in recombinant strains *luxS*-pMG76e-L-ZS9, pMG76e-L-ZS9, and the wild-type strain. As shown in Figure 3, introduction of empty plasmid pMG76e had no influence on AI-2 activities of L-ZS9 from 2 to 24 h. The AI-2 activities of *luxS*-pMG76e-L-ZS9 were significantly increased compared to pMG76e-L-ZS9 and the wild-type strain from 6 to 24 h. The AI-2 activity of *luxS*-pMG76e-L-ZS9 was almost double that of pMG76e-L-ZS9 and wild-type strain in the cell-free culture fluid (CF) collected at 24 h. These results suggested that overexpression of *luxS* gene promoted AI-2 production in *L. paraplantarum* L-ZS9.

### Overexpression of *luxS* gene had no influence on the growth of *L. paraplantarum* L-ZS9

**Figure 4:**
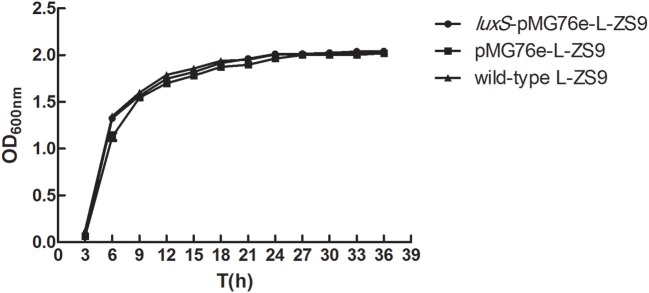
Growth curves of pMG76e-L-ZS9, *luxS*-pMG76e-L-ZS9 and wild-type strains. Data are presented as mean ± SEM. *n* ≥ 3.

In order to determine whether overexpression of luxS influenced the growth of L-ZS9, we evaluated bacterial growth by measuring OD_600_ from 3 to 36 h. Overexpression of wild-type L-ZS9 with *luxS* showed a comparable growth rate to that of pMG76e-L-ZS9 (Figure 4). These data suggested that overexpression of *luxS* gene exerts no significant effect on the growth of L-ZS9.

### Overexpression of *luxS* gene enhanced biofilm formation of *L. paraplantarum* L-ZS9

**Figure 5:**
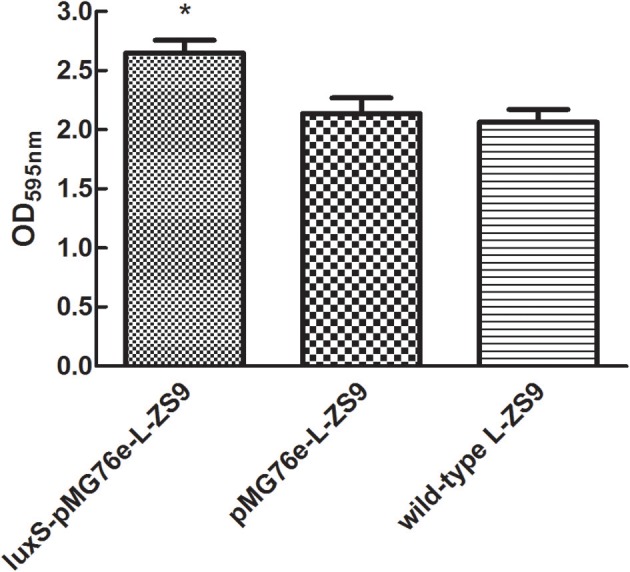
Biofilm formation of pMG76e-L-ZS9, *luxS*-pMG76e-L-ZS9 and wild-type strains. Data are presented as mean ± SEM. *n* ≥ 3. *p < 0.05 compared with the control.

Since LuxS/AI-2-dependent QS exerts essential functions in biofilm formation of many bacterial pathogens in various ways, the ability of recombinant strains to form biofilm was also determined. As shown in Figure 5, there was no significant difference in biofilm formation between pMG76e-L-ZS9 and the wild-type strain, suggesting that the empty plasmid pMG76e had no influence on biofilm formation of L-ZS9. The biofilm formation of luxS-pMG76e-L-ZS9 increased significantly, as compared with the pMG76e-L-ZS9 and the wild-type strain. These data indicate that the LuxS gene was involved in the formation of L-ZS9 biofilms.

### Overexpression of *luxS* gene improved stress resistances of *L. paraplantarum* L-ZS9

**Figure 6:**
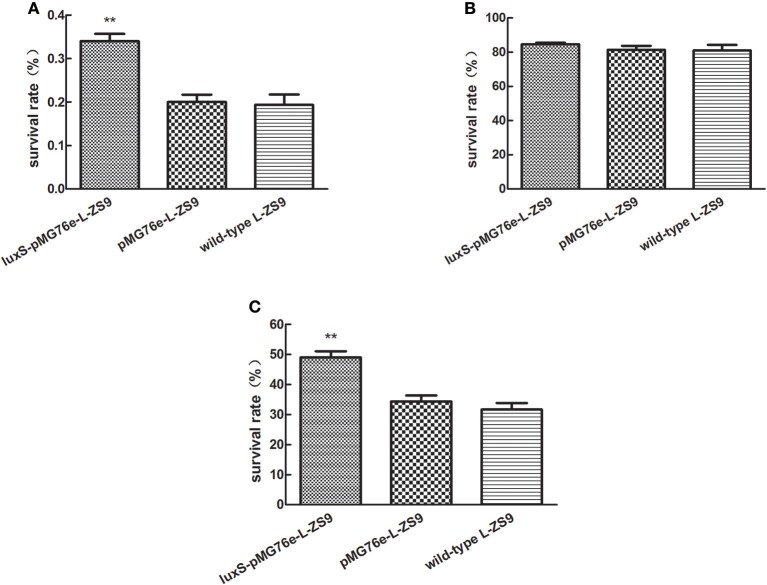
Heat- **(A)**, acid- **(B)**, and bile salt- **(C)** resistance of pMG76e-L-ZS9, *luxS*-pMG76e-L-ZS9, and wild-type strains. Data are presented as mean ± SEM. *n* ≥ 3. **p < 0.01 compared with the control.

In nature, bacteria have developed a variety of mechanisms of resistance to environmental stresses. Since biofilm formation has been reported to contribute to stress resistance of bacteria, in order to determine the role of LuxS in stress resistance of L-ZS9, we tested the survival in heat, acid and bile salt. As shown in Figure 6, there was no significant difference in heat-, acid- and bile resistance between pMG76e-L-ZS9 and the wild-type strain, suggesting that the empty plasmid pMG76e had no influence on stress resistance of L-ZS9. However, the heat- and bile- resistance activities but not acid-resistance of *luxS*-pMG76e-L-ZS9 were much higher than that of pMG76e-L-ZS9 and the wild-type strain (Figures 6A,C). The survival rates of *luxS*-pMG76e-L-ZS9 were about 179 and 153% with the wild-type as 100% after the heat- and bile- shock, respectively. Considering the initial cell number was 1 × 10^9^, the increased amounts of *luxS*-pMG76e-L-ZS9 were 1.5 × 10^6^ and 1.7 × 10^8^ after the heat- and bile-shock, respectively. These results suggested that LuxS was contribute to the resistance activities of *L. paraplantarum* L-ZS9 against heat and bile salt.

### *luxS* overexpression altered gene transcription profiles in *L. paraplantarum* L-ZS9

**Figure 7:**
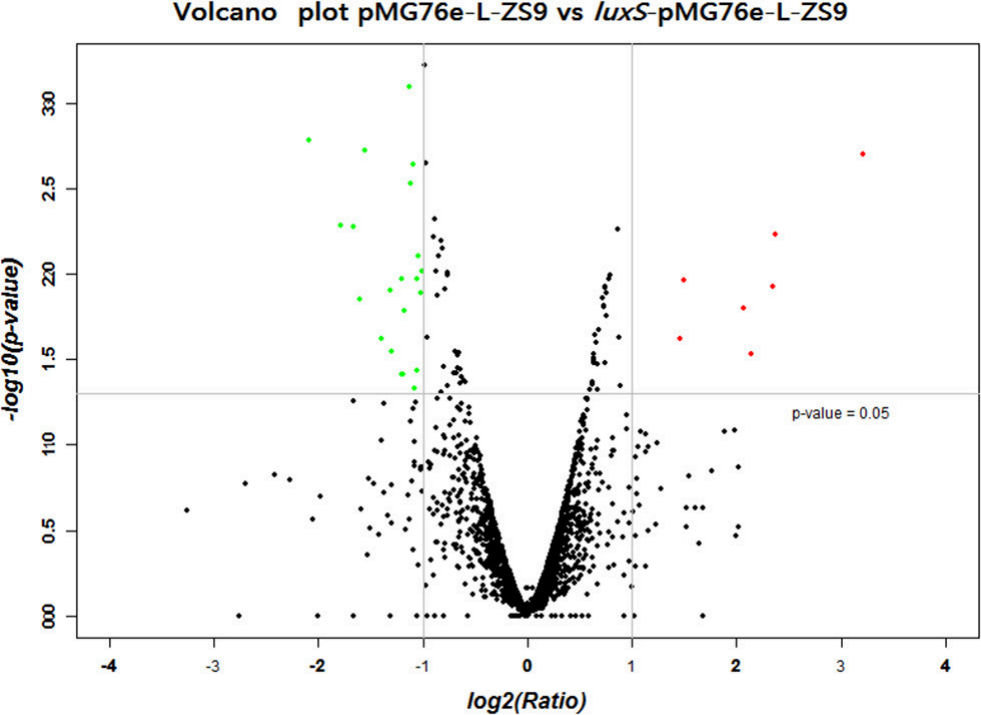
Volcano plot of different expression genes of pMG76e-L-ZS9 and *luxS*-pMG76e-L-ZS9 strains.

In order to identify genes that were regulated by *luxS*, comparative RNA-seq was carried out in both recombinant strains *luxS*-pMG76e-L-ZS9 and pMG76e-L-ZS9. The concentration of the extracted RNA was shown in Table S1 and their agrose gel eletrophoresis images were shown in Figure S1. Their raw and filtered data quality was listed in Table S2. The evaluation of data quality of pMG76e-L-ZS9 and *luxS*-pMG76e-L-ZS9 was shown in Figures S2, S3. In this study, 35 differentially expressed genes (change at least 2-folds) were identified (Table 1) and their volcano plot was shown in Figure 7. Among these genes, gene *luxS* was up-regulated by 205 folds. In addition, the expression of 12 genes encoding hypothetical proteins, 9 genes encoded transporter proteins and 3 genes encoded membrane proteins was altered, which totally accounted for about 50% of annotated genes.

**Table 1:** Differentially expressed genes in cells of *luxS*-pMG76e-L-ZS9 compared to pMG76e-L-ZS9.

**Gene name**	**Fold change (log2)**	**ID**	**Protein ID**	**Product**
ASU28_RS14150	inf	gene2829	WP_033611588.1	Allulose-6-phosphate 3-epimerase
ASU28_RS08065 *sugE*	inf	gene1612	WP_033610729.1	QacE family quaternary ammonium compound efflux SMR transporter
ASU28_RS10630	inf	gene2125	WP_033612003.1	Hypothetical protein
Hypothetical protein	inf	gene2744	
ASU28_RS05215	inf	gene1042	WP_021731301.1	Hypothetical protein
ASU28_RS07600	inf	gene1519	WP_003640551.1	Hypothetical protein
ASU28_RS04530 *luxS*	7.68443	gene905	WP_003641031.1	S-ribosylhomocysteine lyase
ASU28_RS09300	1.49615	gene1859	WP_033610969.1	Hypothetical protein
ASU28_RS11200 *betT*	1.4642	gene2239	WP_033612092.1	BetT protein
ASU28_RS07105	−1.00272	gene1420	WP_021731448.1	Integral membrane protein
ASU28_RS03050	−1.01778	gene609	WP_033610853.1	Nitrobenzoate reductase
ASU28_RS12410	−1.04367	gene2481	WP_033610593.1	AraC family transcriptional regulator
ASU28_RS11675	−1.05829	gene2334	WP_033610057.1	DNA-binding protein
ASU28_RS04420	−1.05901	gene883	WP_033611045.1	Phosphate ABC transporter ATP-binding protein
ASU28_RS14105	−1.07651	gene2820	WP_033611599.1	PTS beta-glucoside transporter subunit EIIBCA
ASU28_RS10550 *recT*	−1.09727	gene2109	WP_033611994.1	DNA recombination protein RecT
ASU28_RS10555	−1.09727	gene2110	WP_033611995.1	Hypothetical protein
ASU28_RS06500	−1.11328	gene1299	WP_021731687.1	Hypothetical protein
ASU28_RS05530	−1.1354	gene1105	WP_033609957.1	Membrane protein
ASU28_RS05645	−1.17751	gene1128	WP_021731360.1	Hypothetical protein
ASU28_RS01930	−1.18473	gene385	WP_033611287.1	Glycerol transporter
ASU28_RS12280	−1.1997	gene2455	WP_033610185.1	DNA-3-methyladenine glycosylase
ASU28_RS05640	−1.20425	gene1127	WP_021731359.1	MATE family efflux transporter
ASU28_RS03805	−1.30129	gene760	WP_021731968.1	Hypothetical protein
ASU28_RS03675	−1.31353	gene734	WP_033610025.1	Glycine cleavage system protein H
ASU28_RS01640	−1.39723	gene327	WP_033610437.1	Hypothetical protein
ASU28_RS10560	−1.55545	gene2111	WP_033611996.1	Hypothetical protein
integrase	−1.6056	gene2702	
ASU28_RS03705	−1.66843	gene740	WP_033610031.1	GNAT family acetyltransferase
ASU28_RS10520	−1.78945	gene2103	WP_052048925.1	Transcriptional regulator
ASU28_RS03605 *nhaC*	−2.08869	gene720	WP_033610935.1	Na+/H+ antiporter NhaC
ASU28_RS08220	inf	gene1643	WP_033610753.1	PTS sugar transporter subunit IIA
ASU28_RS10765	inf	gene2152	WP_033612020.1	Hypothetical protein
ASU28_RS03720	inf	gene743	WP_033610033.1	Membrane protein
ASU28_RS08120	inf	gene1623	WP_033610736.1	Membrane protein

*inf, infinite*.

## Discussion

LuxS is a key enzyme for the biosynthesis of AI-2. The precursor of AI-2, 4,5-dihydroxy-2,3 pentanedione (DPD), is a by-product of methionine catabolism formed through three reactions. The first step for AI-2 formation is the removal of methyl group from S-adenosylmethionine (SAM), which is catalyzed by SAM-dependent methylferases. The resulting product, S-adenosylhomocysteine (SAH), is converted to S-ribosylhomocysteine (SRH) by the enzyme SAH nucleosidase (Parveen and Cornell, 2011). SRH is hydrolysed to DPD by LuxS (Schauder et al., 2001). DPD further undergoes autocatalytically hydrolysis to form AI-2. Many studies have pointed out the pivotal role of LuxS in biological processes in many bacteria. Our previous data found that D-Ribose interferes with quorum sensing to inhibit biofilm formation of *L. paraplantarum* L-ZS9, suggesting the significance of quorum sensing in biological activity of L-ZS9. In this study, we have identified the role for LuxS as a positive regulator of biofilm formation and stress resistance in *L. paraplantarum* L-ZS9. To examine the effect of LuxS on the biological process of *L. paraplantarum*, we constructed a LuxS overexpressed L-ZS9 strain. The recombinant strain *luxS*-pMG76e-L-ZS9 displayed enhanced AI-2 production, along with that of previous published studies in other bacteria, clearly indicating that LuxS contributes to AI-2 synthesis in *L. paraplantarum*. It is interesting that manipulation of AI-2 levels alters the composition of the animal gut microbiota. The *E. coli* strain overproducing AI-2 changes the balance between *Firmicutes* and *Bacteroidetes* in the gut of a streptomycin-treated mouse, partly offsetting the effects of the antibiotic (Sourjik and Vorholt, 2015). Therefore, probiotics overproducing AI-2 may have better effects on regulation of gastrointestinal microbiota and their beneficial functions need further investigation.

Previous studies have reported that LuxS acts in concert to control the adaption of *A. actinomycetemcomitans* to iron-limiting conditions and its growth under such conditions (Fong et al., 2003). Deletion of *luxS* in *B. anthracis Sterne* strain 34F2 results in inhibition of AI-2 synthesis and a growth defect (Jones et al., 2016). However, *luxS* gene does not regulate the growth rate *E. faecalis*, but affects the biofilm formation (He et al., 2016). The data we present here, along with that of *E. faecalis*, suggested that overexpression of *luxS* did not affect the L-ZS9 growth. LuxS is involved in biofilm formation of various strains (Sela et al., 2006; Karim et al., 2013; Niu et al., 2013; He et al., 2015). The luxS mutants of L. rhamnosus GG have a lower capacity to form biofilms than the wild-type strain (Lebeer et al., 2007, 2008). In consistence with these studies, LuxS was involved in biofilm formation of L-ZS9 and its overexpression enhanced the biofilm formation. Biofilms constitute the predominant microbial style of life in natural and engineered ecosystems. The formation of biofilms, microbial communities embedded in self-produced polymeric matrices attached to a surface, is an ancient and universal trait that enables microorganisms to develop coordinated architectural and survival strategies (Gambino and Cappitelli, 2016). Biofilms are closely related with adhesion. This life style helps microorganisms to survive under unfavorable environmental conditions (Taylor et al., 2014; Olsen, 2015). It has been demonstrated that biofilms of *Lactobacillus* exhibit higher tolerance than planktonic counterpart (Kubota et al., 2009; Cheow and Hadinoto, 2013). The biofilm enhances the immunomodulatory effects of *Lactobacillus* sp. (Rieu et al., 2014). It has been reported that the *luxS* gene plays an important role in the gastrointestinal environment tolerance and adhesion ability of *Lactobacillus plantarum* KLDS1.0391 (Jia et al., 2018). AI-2 inhibition correlated with a reduction in the stress-related genes of *Lactobacillus rhamnosus* (Yeo et al., 2015). In addition, LuxS metabolism is crucial for the gastric stress resistance of *L. rhamnosus* GG (Lebeer et al., 2008). In this study, *luxS*-pMG76e-L-ZS9 had greater biofilm formation ability, suggesting that the recombinant strain may have greater resistance to adverse stress.

It has been reported that quorum sensing is involved in regulating drug resistance of bacteria (Liang et al., 2016; Xue et al., 2016; Yufan et al., 2016; Lai et al., 2017), but few studies have investigated the regulation of stress resistance by quorum sensing or LuxS. Quorum sensing facilitates the stress resistance of *E. coli* in microcapsules by detecting the expression of *luxS*/AI-2 system (Gao et al., 2016). In this study, the heat- and bile- resistances of L-ZS9 were significantly improved when *luxS* was overexpressed in the strain. How LuxS gene regulates stress resistance of L-ZS9 in this context remains to be fully elucidated, but is likely to involve the changed gene expression profile.

Identification of gene expression profile at the presence of LuxS may have important industrial implications. RNAseq analysis identified 35 differentially expressed genes in recombinant strains *luxS*-pMG76e-L-ZS9. Multiple efflux systems are classified into five different families, including ATP-binding cassette (ABC) family, major facilitator super family (MFS), resistance/nodulation/cell division (RND) family, multidrug, and toxic—compound extrusion (MATE) family and the small multidrug resistance (SMR) family. In the present study, some members of ABC, MATE, and SMR families were identified to be regulated by *luxS*. There are three main mechanisms playing pivotal roles in cell survival with biofilms: biofilm-specific protection against oxidative stress, the expression of efflux pumps and the protection provided by matrix polysaccharides (Billings et al., 2015; Jhajharia et al., 2015; Rasamiravaka et al., 2015). It has been reported that biofilm formation of *E. coli* was regulated by many redundant multidrug resistance transporters (Bay et al., 2016). One SMR transporter, SugE, was identified in *luxS*-pMG76e-L-ZS9 strain. SugE is a suppressor of *groEL* mutation proteins and they may play an important role in the uptake of chaperone regulatory compounds (Bay and Turner, 2011). And gene *sugE* affected biofilm formation by modulating capsular polysaccharide (CPS) production in a *Klebsiella pneumoniae* strain (Wu et al., 2011). Two MATE genes (ASU28_RS05640 and ASU28_RS03605) were down-regulated in *luxS*-pMG76e-L-ZS9. MATE efflux transporters are believed to be universally present in all living organisms. They play critical roles in cellular physiology and drug resistance (Kuroda and Tsuchiya, 2009). ABC transporters are integral membrane proteins that move diverse substrates across cellular membranes (Hopfner, 2016). And it has been reported that the signal AI-2 could be imported through ABC type importers (Quan et al., 2016). In addition, two genes of phosphotransferase system (PTS) were identified. Gene ASU28_RS14105 was down-regulated and ASU28_RS08220 did not expressed in *luxS*-pMG76e-L-ZS9. PTS is a sugar-phosphorylating system and a complex protein kinase system that regulates a wide variety of metabolic processes and controls the expression of numerous genes (Khajanchi et al., 2015; Somavanshi et al., 2016; Westermayer et al., 2016). It has been reported that AI-2 could be imported through the PTS (Quan et al., 2016). These results suggested that *luxS* regulated expression of membrane and transporter related proteins.

In the present study, one AraC family of transcriptional regulator (ASU28_RS12410) was differentially expressed. The AraC family of transcriptional regulators (AFTRs) constitutes one of the largest groups of regulatory proteins in bacteria. It has been reported that AraC-type transcriptional regulator Rbf plays an important role in biofilm-associated medical-device-related infection (Rowe et al., 2016). In addition, gene ASU28_RS01930 was down-regulated in *luxS*-pMG76e-L-ZS9. This gene encodes glycerol transporter/aquaporin/glycerol uptake facilitator protein. It has been reported that aquaporin 7 is an aquaglyceroporin that has been found to operate as a facilitative carrier rather than a channel for glycerol, although its primary function is as a water channel (Katano et al., 2014). This study showed that multiple gene or proteins including transporters and transcriptional regulator and other proteins were regulated by *luxS*.

## Conclusions

**Figure 8:**
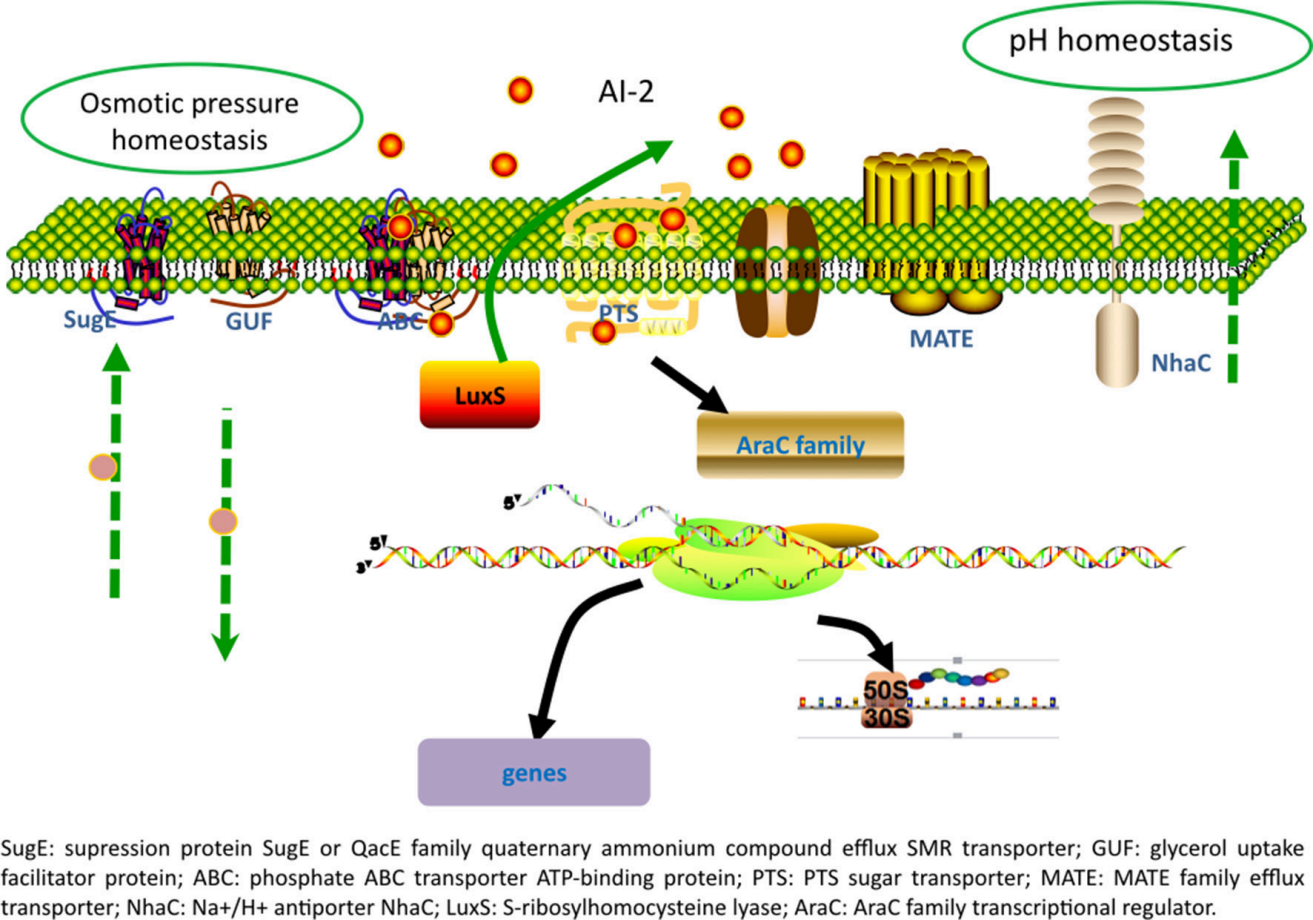
A hypothetical model of the effect of LuxS overexpression on *L. paraplantarum* L-ZS9.

Overexpression of *luxS* in *L. paraplantarum* L-ZS9 promoted AI-2 production and enhanced the stress-resistance and biofilm formation of this strain. As shown in Figure 8, *luxS* plays roles in modulating physiological behaviors of this strain mainly through regulating multiple transporters and transcriptional regulator and the underlying mechanism needs to be further elucidated.

## Author contributions

LL and PL conceived and designed the experiments. LL performed the experiments. LL and RW analyzed the data. JZ contributed reagents. LL wrote the paper.

### Conflict of interest statement

The authors declare that the research was conducted in the absence of any commercial or financial relationships that could be construed as a potential conflict of interest.

## Supplementary material

Table S1 Concentration of RNA of pMG76e-L-ZS9 and *luxS*-pMG76e-L-ZS9 strains.

Table S2 Raw and filtered data quality of pMG76e-L-ZS9 and *luxS*-pMG76e-L-ZS9.

Figure S1 Agrose gel electrophoresis of RNA of pMG76e-L-ZS9 and *luxS*-pMG76e-L-ZS9 strains.

Figure S2 Evaluation of data quality of pMG76e-L-ZS9.

Figure S3 Evaluation of data quality of *luxS*-pMG76e-L-ZS9.
