# Supplementary material for: Overexpression of luxS Promotes Stress Resistance and Biofilm Formation of Lactobacillus paraplantarum L-ZS9 by Regulating the Expression of Multiple Genes
Source: Front Microbiol. 2018 Nov 12;9:2628. doi: 10.3389/fmicb.2018.02628 (PMC6240686; doi:10.3389/fmicb.2018.02628)
Supplement: Table S1 — Concentration of RNA of pMG76e-L-ZS9 and luxS-pMG76e-L-ZS9 strains. [file Table_1.DOC]

Table S1. Concentration of RNA of pMG76e-L-ZS9 and *luxS*-pMG76e-L-ZS9 strains

| Sample name | Concentration（ng/μL） | OD260/280 |
| --- | --- | --- |
| pMG76e-L-ZS9 RNA | 2332.0 | 2.04 |
| *luxS*-pMG76e-L-ZS9 RNA | 1933.0 | 2.10 |
