# Supplementary material for: Overexpression of luxS Promotes Stress Resistance and Biofilm Formation of Lactobacillus paraplantarum L-ZS9 by Regulating the Expression of Multiple Genes
Source: Front Microbiol. 2018 Nov 12;9:2628. doi: 10.3389/fmicb.2018.02628 (PMC6240686; doi:10.3389/fmicb.2018.02628)
Supplement: Table S2 — Raw and filtered data quality of pMG76e-L-ZS9 and luxS-pMG76e-L-ZS9. [file Table_2.DOC]

Table S2. Raw and filtered data quality of pMG76e-L-ZS9 and *luxS*-pMG76e-L-ZS9

| Sample  Name | Read-  Length | Raw-  Read | Filter-  Read | Filter-  Read（%） | Raw-  Base | Filter-  Base | Q20  (%) |
| --- | --- | --- | --- | --- | --- | --- | --- |
| pMG76e-  L-ZS9 | 150 | 26571550 | 25865172 | 97.34 | 3.99 G | 3.95 G | 98.98 |
| *luxS*-pMG76e-L-ZS9 | 150 | 30573377 | 29685478 | 97.1 | 4.59 G | 4.54 G | 98.97 |
