# Supplementary figures and images for: Overexpression of luxS Promotes Stress Resistance and Biofilm Formation of Lactobacillus paraplantarum L-ZS9 by Regulating the Expression of Multiple Genes
Source: Front Microbiol. 2018 Nov 12;9:2628. doi: 10.3389/fmicb.2018.02628 (PMC6240686; doi:10.3389/fmicb.2018.02628)

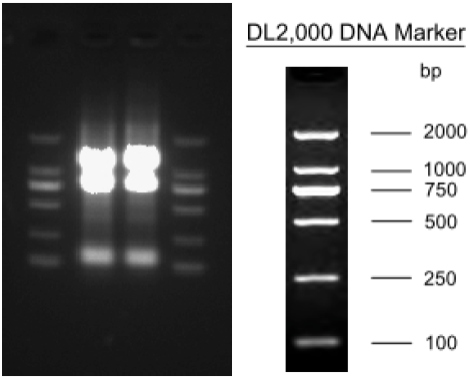

Supplement: Figure S1 — Agrose gel electrophoresis of RNA of pMG76e-L-ZS9 and luxS-pMG76e-L-ZS9 strains. [file Image_1.TIF]

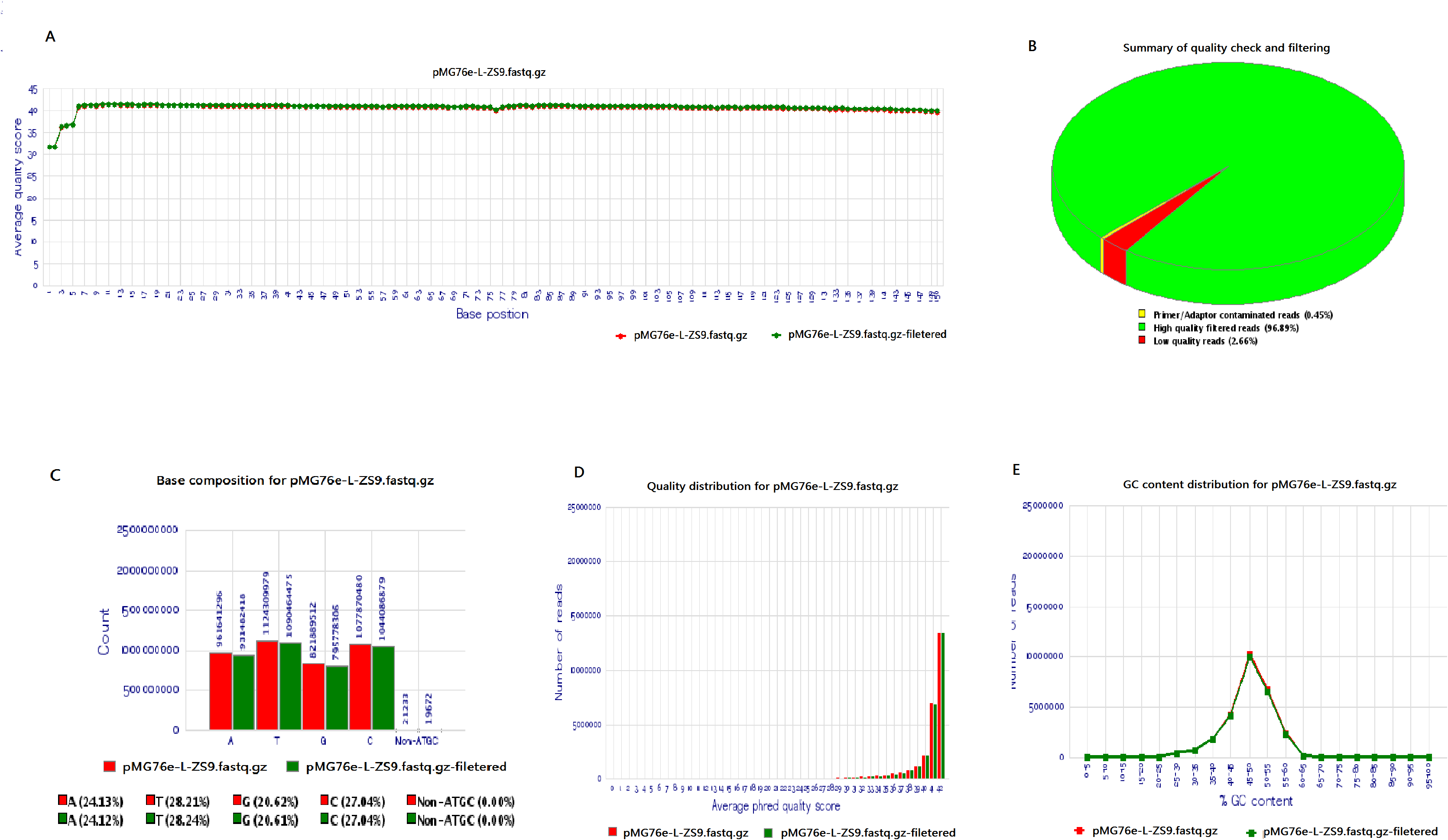

Supplement: Figure S2 — Evaluation of data quality of pMG76e-L-ZS9. [file Image_2.TIF]

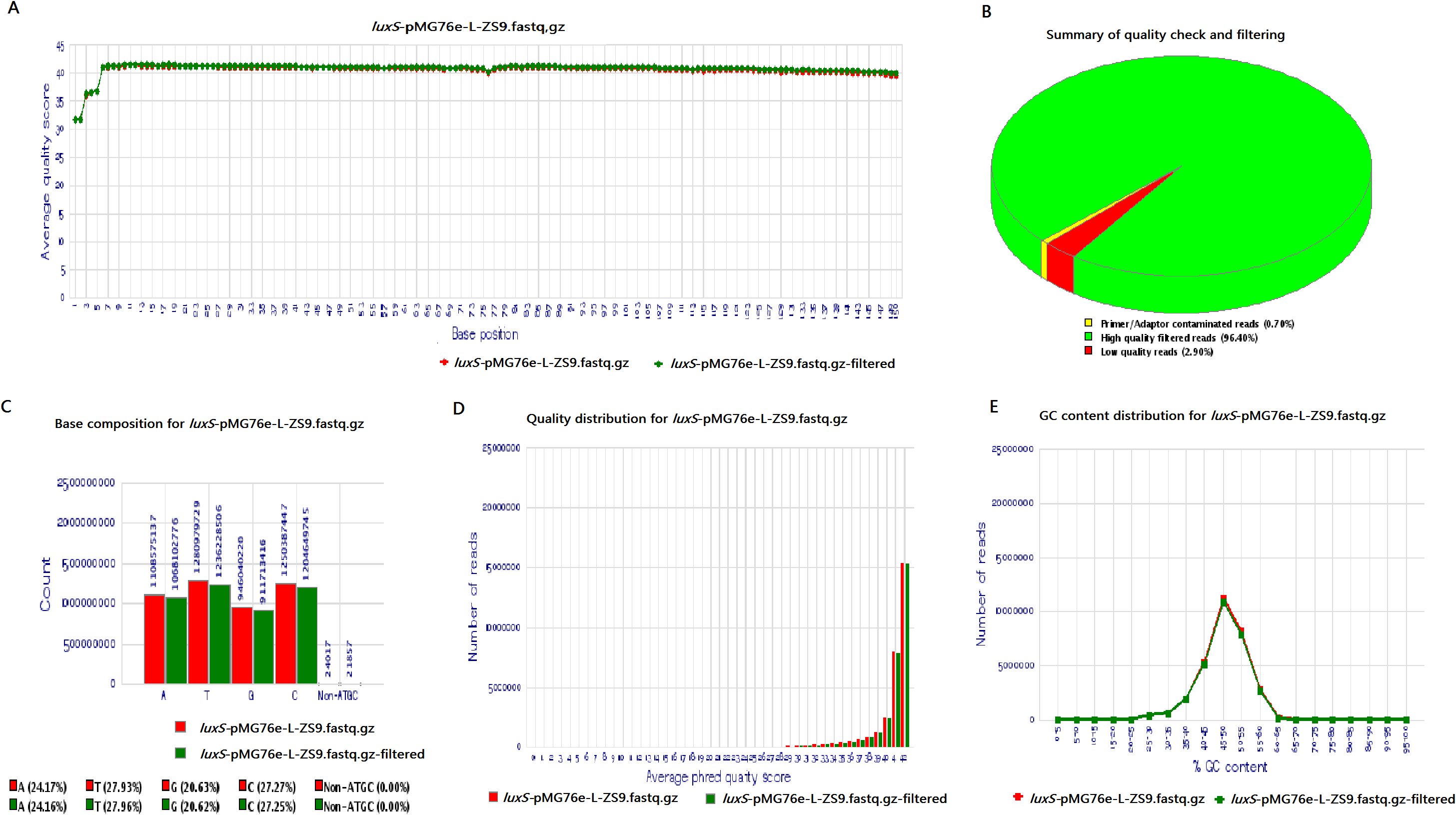

Supplement: Figure S3 — Evaluation of data quality of luxS-pMG76e-L-ZS9. [file Image_3.TIF]
